# Improvement of Internal Tumor Volumes of Non-Small Cell Lung Cancer Patients for Radiation Treatment Planning Using Interpolated Average CT in PET/CT

**DOI:** 10.1371/journal.pone.0064665

**Published:** 2013-05-16

**Authors:** Yao-Ching Wang, Hsun-Lin Tseng, Yang-Hsien Lin, Chia-Hung Kao, Wei-Chien Huang, Tzung-Chi Huang

**Affiliations:** 1 Division of Radiation Oncology, China Medical University Hospital, Taichung City, Taiwan; 2 Department of Biomedical Imaging and Radiological Science, China Medical University, Taichung City, Taiwan; 3 Graduate Institute of Clinical Medical Science, China Medical University, Taichung City, Taiwan; 4 Department of Nuclear Medicine, China Medical University Hospital, Taichung City, Taiwan; 5 Graduate Institute of Cancer Biology, China Medical University, Taichung City, Taiwan; Dresden University of Technology, Germany

## Abstract

Respiratory motion causes uncertainties in tumor edges on either computed tomography (CT) or positron emission tomography (PET) images and causes misalignment when registering PET and CT images. This phenomenon may cause radiation oncologists to delineate tumor volume inaccurately in radiotherapy treatment planning. The purpose of this study was to analyze radiology applications using interpolated average CT (IACT) as attenuation correction (AC) to diminish the occurrence of this scenario. Thirteen non-small cell lung cancer patients were recruited for the present comparison study. Each patient had full-inspiration, full-expiration CT images and free breathing PET images by an integrated PET/CT scan. IACT for AC in PET_IACT_ was used to reduce the PET/CT misalignment. The standardized uptake value (SUV) correction with a low radiation dose was applied, and its tumor volume delineation was compared to those from HCT/PET_HCT_. The misalignment between the PET_IACT_ and IACT was reduced when compared to the difference between PET_HCT_ and HCT. The range of tumor motion was from 4 to 17 mm in the patient cohort. For HCT and PET_HCT_, correction was from 72% to 91%, while for IACT and PET_IACT_, correction was from 73% to 93% (*p<0.0001). The maximum and minimum differences in SUV_max_ were 0.18% and 27.27% for PET_HCT_ and PETIACT, respectively. The largest percentage differences in the tumor volumes between HCT/PET and IACT/PET were observed in tumors located in the lowest lobe of the lung. Internal tumor volume defined by functional information using IACT/PET_IACT_ fusion images for lung cancer would reduce the inaccuracy of tumor delineation in radiation therapy planning.

## Introduction

PET/CT combines F^18^-FDG positron emission tomography (PET), and computed tomography (CT) images with both functional and anatomic information provide a more precise diagnostic reference for tumor volume, tumor locations and tumor staging. Therefore, PET/CT has been increasingly used for target volume delineation in radiotherapy treatment planning (RTP) to deliver the optimal radiation dose to tumors and to decrease the radiation dose to surrounding normal tissues [1]–[3]. The reduction of intra- and inter-observer variability in target volume delineation by contouring with PET/CT has also been reported in previous studies [4]–[5]. Moreover, PET is a useful imaging tool to differentiate between inflammation and malignance, such as lung atelectasis, mediastinal lymphadenopathy, and distant metastases [6]. The incorporation of PET information in RTP along with the CT-based gross tumor volume can improve the definition of tumor volume and has been extensively used in radiotherapy.

Respiration produces additional variation in imaging diagnosis and target contouring for radiotherapy in thoracic malignancies. CT can provide high spatial information and attenuation correction for PET in PET/CT. However, respiratory motion causes uncertainties in the tumor edges on either CT or PET images and causes misalignment when registering PET and CT images. Consequently, this phenomenon may influence oncologists when attempting to delineate tumor volume accurately in RTP [3], [7]–[9].

Many studies have improved the misalignment in PET/CT fusion image due to respiration by using gated (4D) imaging techniques [3], [10]–[12]. With gated technology aids, the reduction of motion artifacts and increases in the accuracy of tumor volume and localization delineation when compared to non-gated PET were achieved. Moreover, we previously proposed an interpolation method with interpolated average CT (IACT) for attenuation correction (AC) to reduce the PET/CT misalignment [12]–[13]. Using IACT imaging, standardized uptake value (SUV) can be corrected with a lower radiation dose compared to the use of gated imaging. In the present study, the comparison of tumor volumes for RTP is reported. We assess the differences in tumor volumes between 3D PET/CT and PET/IACT and evaluate the SUVmax changes in terms of tumor locations.

## Materials and Methods

### Patient selection

With IRB approval (DMR98-IRB-171-1) for the application of 4D PET/CT to tumor delineation in RTP, thirteen non-small cell lung cancer patients were recruited for this comparison study. All patients signed written, informed consent. There were 9 tumors in the upper lobe and 4 tumors in the lower lobe. The clinical characteristics are summarized in Table 1.

**Table 1:** List of clinical characteristic for all patents.

Patient	Gender	Age	Histology	Tumor location	Stage
1	M	79	Squamous cell carcinoma	LUL	III
2	F	51	Adenocarcinoma	RUL	I
3	M	50	Large cell carcinoma	RUL	III
4	F	74	Adenocarcinoma	RUL	I
5	M	60	Adenocarcinoma	RUL	II
6	M	67	Adenocarcinoma	RUL	IV
7	F	52	Adenocarcinoma	LUL	III
8	F	52	Adenocarcinoma	RUL	III
9	M	62	Adenocarcinoma	LUL	III
10	M	74	Squamous cell carcinoma	LLL	I
11	F	56	Adenocarcinoma	LLL	I
12	M	61	Adenocarcinoma	RLL	III
13	F	61	Adenocarcinoma	RLL	I

Abbreviations — LUL  =  Left Upper Lobe, RUL  =  Right Upper Lobe, LLL  =  Left Lower Lobe, RLL  =  Right Lower Lobe.

### PET/CT

FDG-PET/CT scans were obtained for tumor staging work-ups before cancer treatment. All patients had undergone the standard procedure of PET/CT (PET/CT-16 slice, Discovery STE, GE Medical System, Milwaukee, Wisconsin USA) scanning. Patients were injected with 370 MBq of 18F-FDG and rested during the pharmacokinetics uptake period. The original data included a series of helical CTs (HCT), two extreme phases CTs that considered the full-expiration and the full-inspiration CTs and a whole body PET. HCT images obtained with 120 kVp, variable mA (30–210 mA), 1.75∶1 pitch, 8×3.75 mm x-ray collimation, and 0.5 gantry rotation time and gated CTs were acquired under the same conditions. PET data were acquired at the 3 min per 15 cm bed position; attenuation corrections of PET_HCT_ and PET_IACT_ utilized HCT and IACT, respectively.

### Tumor motion and interpolated average CT

**Figure 1:**
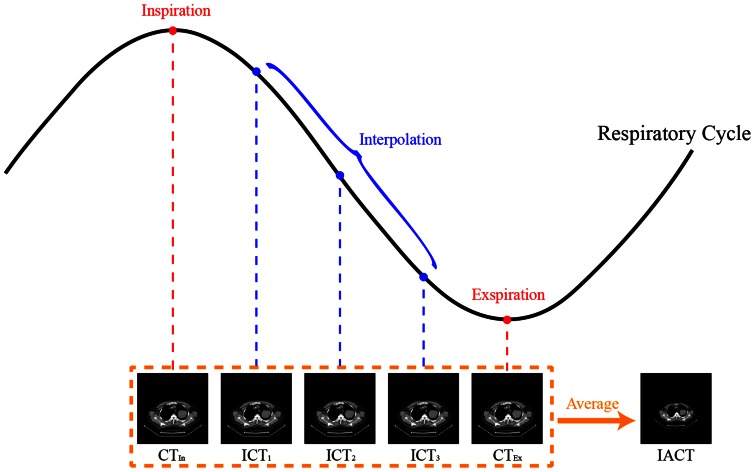
Illustrates the generation of IACT from full-inspiration and full-expiration images by OFM. The resulted deformation matrix is then used to interpolate the phases in between with 4 equal spatial steps. The IACT is the average of the two original phases and the interpolated 3 phases (ICTs) for attenuation correction in PET reconstruction.

We previously proposed an IACT method from 4D-CT in comparison with 4D-Cine CT [12]. IACT is a robust, accurate low dose alternate to CACT and works well for a large range of respiratory motion amplitudes, as was reported in a simulation study [13]. The full-inspiration and full-expiration CT sets as two-extreme-phase images were used to generate the motion maps using the optical flow method (OFM), a deformable image registration algorithm [14]. The total motion range for each voxel in the forward motion map is equally spaced into 4 intervals, resulting in 3 sets of interpolated CT (ICT) image sets as the mid-phases from inspiration to expiration. (Figure 1). The 3 interpolated phases together with the two original phases, including one inhalation and expiration, compose a complete respiratory cycle. These 5 phases are averaged to generate the IACT for AC on PET data. The conclusion that the radiation dose using IACT could be reduced by 85% compared to that of 4D-CT was reported in previous study [12]–[13]. IACT serves as a low-dose alternative to 4D-CT. The OFM calculation was the following:
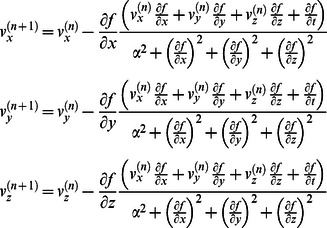
where n is the number of iterations, *v* ^(n)^ is the average velocity driven from the surrounding voxels, *f(x, y, z, t)* is the differentiable image intensity at position *(x, y, z)* at time *t*, and α is the weighting factor with an empirical value of 5. The given equations are applied to estimate the displacement for tumor motion between full-expiration and full-inspiration.

### Tumor volume analysis

An experienced radiation oncologist manually delineated the internal tumor volume (TV) for all patients on HCT, IACT with fusion images of the individual PET_HCT_ and PET_IACT_. Tumor volumes from HCTs (TV_HCT_) and from IACTs (TV_IACT_) consisted of the control group and the experiment group. Tumor volumes delineated with CT using CT/PET fusion information were compared with the percentage difference, which was calculated by the equation 
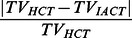
. We also assessed the similarity between HCT/PET_HCT_ and IACT/PET_IACT_ fusion images using the ratio of the intersection to the union of TV. The correlation for compared TVs is defined as 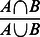, where A and B are the different tumor volumes from CT-based and PET-based images [15]. The metabolic rate of glucose, SUV_max_ from FDG-PET, was also applied to represent the physiology information within the tumor volume.

## Results

**Figure 2:**
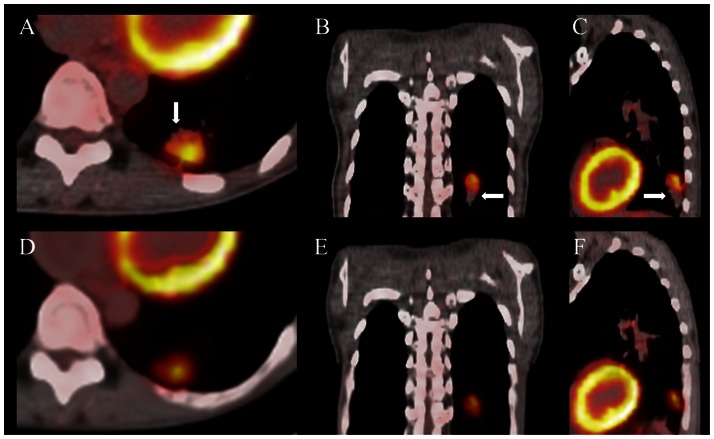
Represents the PET/CT fusion for tumor contour delineation. The arrows indicate the mismatch observed in PET_HCT_/HCT fusion between PET_HCT_ and HCT, showing the (A) transverse (B) coronal and (C) sagittal view. The image fusion with PET_IACT_ and IACT is seen in the (D) transverse (E) coronal and (F) sagittal view.

Figure 2 shows the PET/CT fusion for tumor contour delineation. The arrows indicate the mismatch observed in PET_HCT_/HCT fusion between PET_HCT_ and HCT showing in the (A) transverse (B) coronal and (C) sagittal views. The correct image fusion with misalignment reduction on PET_IACT_ and IACT represents the (D) transverse (E) coronal and (F) sagittal view. Table 2 shows the gross tumor volumes of HCT/PET_HCT_ and IACT/PET_IACT_, along with estimation of the tumor motion, and the correction of HCT with PET_HCT_ and IACT with PET_IACT._ The median (range) tumor volume for HCT/PET was 31 (4–169) mL, while for the corresponding IACT/PET_IACT_, the median tumor volume was 26 (3–149). The median tumor difference for HCT/PET_HCT_ and IACT/PET_IACT_ was 14% higher (range 5–24%). The range of tumor motion was from 4 to 17 mm. HCT and PET_HCT_ correction was from 72% to 91%, while IACT and PET_IACT_ was from 73% to 93% (*p<0.0001). Table 3 shows the SUV_max_ measurement for each tumor from PET_HCT_ and PET_IACT_. The median SUV_max_ was about 9.65 (1.98–8.77) and 9.53 (1.97–18.59) for PET_HCT_ and PETIACT, respectively. The maximum and minimum differences among SUV_max_ were 0.18% and 27.27%, respectively.

**Table 2:** Tumor volumes and correlation.

	TV (mL)				Correlation (%)	
Patient	HCT/PET_HCT_	IACT/PET_IACT_	Diff. %	Tumor motion (mm)	HCT & PET_HCT_*	IACT & PET_IACT_*
1	73	62	14	9	82	85
2	31	26	14	8	82	88
3	158	150	5	4	91	93
4	26	23	12	7	78	80
5	29	26	9	3	84	89
6	4	3	5	8	77	78
7	99	86	13	10	81	90
8	11	10	6	4	83	88
9	169	149	11	5	82	88
10	7	5	19	12	76	79
11	11	8	24	10	74	74
12	92	76	17	12	79	80
13	127	100	21	17	72	73

Abbreviations – Diff. %  =  percentage difference, HCT  =  Helical CT, IACT  =  Interpolated Average CT, PET_HCT_  =  Attenuation correction of PET image with HCT, PET_IACT_  =  Attenuation correction of PET image with IACT. *p<0.0001.

**Table 3:** SUV_max_ for tumor volumes and difference.

Patient	PET_HCT_	PET_IACT_	Diff (%)
1	3.84	3.66	4.68
2	9.65	10.15	1.24
3	10.10	9.53	0.56
4	1.98	1.97	0.50
5	3.53	3.52	0.28
6	11.38	11.58	1.75
7	21.82	21.78	0.18
8	8.31	7.81	0.60
9	18.77	18.59	0.95
10	8.58	6.24	27.27
11	9.53	7.48	21.51
12	11.75	10.73	8.68
13	13.45	11.97	11.00

Abbreviations – PET_HCT_  =  PET image using HCT as attenuation correction, PET_IACT_  =  PET image using HCT as attenuation correction, Diff. % =  percentage difference.

## Discussion

IACT used for attenuation in PET is able to resolve the CT/PET fusion misalignment of thoracic tumors caused by respiration. In this study, we investigated the potential tumor volume delineation using IACT/PET_IACT_ for RTP in Table 2. Compared to tumor volume determined by HCT/PET, larger TVs were observed in all the subjects, and the percentage difference was from 5% to 24%. For PET, imaging is obtained during several breathing cycles and it represents a time-average map. HCT is obtained during a very short period, depending on the machine. The tumor appears in HCT and PET images without considering respiratory motion correction, increasing the uncertainty of the tumor boundaries [3]. Therefore, isotropic extension of the internal tumor volume is usually utilized for adequate coverage of tumors. Considering the tumor contour correction between CTs and PETs, IACT and PET_IACT_ are better correlated than HCT and PET_HCT._ Because IACT/PET_IACT_ aids in defining tumor volume for RTP, tumor volume delineation can be performed more accurately.

The thoracic tumor volume defined by its functional region using PET for RTP is still limited by respiratory motion, which often increases the real tumor size and reduces the SUV. Several studies suggest that gated PET/CT is a better solution for defining the physiological extent of moving tumors and to improve RTP for lung cancers [15]–[16]. In our study, the increase of the SUV_max_ in PET_IACT_ compared to PET_HCT_ was not obvious, as can be seen in Table 3. We found that the reason for this issue was because of the low electron density resulting from the IACT in the averaged ICTs. Because SUV for PET is based on the attenuation coefficient in terms of electron density, the increase in the SUV_max_ within the functional tumor volume was limited.

**Figure 3:**
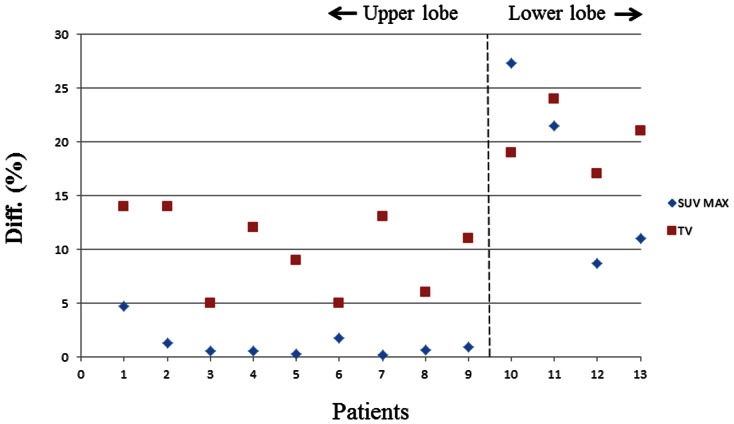
Shows the percentage difference in the tumor volume and the SUV_max_ between delineations from HCT/PET_HCT_ and IACT/PET_IACT_ versus the tumor location inside the lung.

In addition, in Figure 3, we showed the percentage differences of the tumor volume and of SUV_max_ between delineations from HCT/PET_HCT_ and IACT/PET_IACT_ according to tumor location inside the lung. It is intuitively understood that tumors located in the lower lobe of the lung, which is closer to the diaphragm, move more than those in the upper lobe. The larger motion causes more motion blurring in both CT and PET, and therefore, it is possible to extract an inappropriate tumor volume using HCT/PET. Our results show that the percentage difference was more significant for the tumors located in the lower lobe for patents 11–13. A lager respiration motion was observed in the lower lobe of the lung.

There are two limitations to the proposed method. IACT was averaged from the ICTs generated under the equal timing. The ICTs, as with real mid-phase CT, were only generated under the assumption that there was smooth breath from the patients. Second, the full-expiration CT and the full-inspiration CT acquisition can be performed on patients with normal lung function. For patients who are not able to hold their breath for imaging, the proposed interpolating method is limited. In addition, the lack of a ground truth in tumor volume delineation makes difficulty to evaluate the accuracy of the presented IACT method. However, IACT/PET_IACT_ including the respiration information is still superior to the traditional 3D-PET fusion images for lung tumor delineation.

## Conclusion

Our results suggest that tumor volume defined by PET using IACT/PET_IACT_ fusion images for lung cancer would reduce the inaccuracy of tumor delineation compared to using HCT/PET_HCT_.
